# Hospitalization outcomes in patients with schizophrenia after switching to lurasidone or quetiapine: a US claims database analysis

**DOI:** 10.1186/s12913-018-3020-2

**Published:** 2018-04-04

**Authors:** John W. Newcomer, Daisy Ng-Mak, Krithika Rajagopalan, Antony Loebel

**Affiliations:** 1 Charles E. Schmidt College of Medicine, Florida Atlantic University, Boca Raton, FL USA; 2 GHEOR, Sunovion Pharmaceuticals Inc., Marlborough, MA USA; 3 Research & Development, Sunovion Pharmaceuticals Inc., Fort Lee, NJ USA

**Keywords:** Antipsychotic medication, Retrospective studies, Hospital, Schizophrenia

## Abstract

**Background:**

This study compared hospital admission rates among adult patients with schizophrenia who switched to antipsychotic monotherapy with lurasidone or quetiapine.

**Methods:**

This retrospective cohort study used U.S.-based Truven Health MarketScan® Medicaid Multi-State Database (April 2010 through December 2012) and MarketScan® Commercial Claims and Encounters Database (April 2010 through October 2013). Continuous enrollment for 6-months before and after medication initiation was required. Treatment episodes ended after 6-months post lurasidone or quetiapine initiation, a 60-day treatment gap, or initiation of another antipsychotic. Length of treatment episodes (i.e., treatment duration) was compared using a t-test. All-cause, mental-health, and schizophrenia-related hospitalization rates, as well as costs, were compared between lurasidone- and quetiapine-treated patients using multivariable generalized linear models that adjusted for background characteristics.

**Results:**

Quetiapine (*n* = 435) compared to lurasidone (*n* = 238) treatment was associated with increased all-cause (21% vs 13%, *p* < 0.05) and mental health-related hospitalizations (20% vs 12%, p < 0.05), but similar rates of schizophrenia-related hospitalizations (14% vs. 10%, *p* = 0.14). After adjusting for baseline covariates, quetiapine had 64% higher likelihood of all-cause hospitalizations (OR [odds ratio] = 1.64, 95% confidence interval [CI] 1.05–2.57, *p* = 0.03), 74% higher likelihood of mental health-related hospitalizations (OR = 1.74, 95% CI 1.11–2.75, *p* = 0.02), and a similar likelihood of schizophrenia-related hospitalization (OR = 1.35, 95% CI 0.82–2.22, *p* = 0.24). For those with hospital admissions, adjusted all-cause admission costs were higher for quetiapine when compared with lurasidone in both the Medicaid ($22,036 vs. $15,424, *p* = 0.17) and commercial populations ($23,490 vs. $20,049, *p* = 0.61). These differences were not significant. The length of treatment episodes was significantly shorter for quetiapine than lurasidone (115.4 vs 123.1 days, *p* < 0.05).

**Conclusions:**

In this retrospective claims database study, patients with schizophrenia who were switched to lurasidone had significantly fewer all-cause and mental health-related hospitalizations and similar rates of schizophrenia-related hospitalization compared with those switched to quetiapine. Patients switching to lurasidone had a significantly longer treatment duration rate than those switching to quetiapine. These results may reflect differences in efficacy or tolerability between lurasidone and quetiapine.

**Electronic supplementary material:**

The online version of this article (10.1186/s12913-018-3020-2) contains supplementary material, which is available to authorized users.

## Background

Schizophrenia is a chronic, debilitating psychiatric disorder with a lifetime prevalence of approximately 1% [1]. Clinically, schizophrenia is characterized by cognitive impairment, hallucinations, paranoid or bizarre delusions, and social withdrawal resulting in significant social and occupational dysfunction [2–4].

In 2013, the total cost of schizophrenia in the U.S. was estimated at $155.7 billion [5]. Approximately $37.7 billion of the total cost derives from direct healthcare costs, with $15.2 billion of those costs resulting from inpatient care [5–7]. Hospitalizations are a major component of healthcare costs among patients with schizophrenia [6–8]. The risk of hospitalization increases with non-adherence to treatment, and adherence to treatment is often low [9, 10].

Antipsychotics, and in particular atypical antipsychotics, are recommended as the first-line treatment for schizophrenia [11]. Atypical antipsychotics have been found to be a heterogeneous class of drugs with varied efficacy and tolerability profiles [12, 13]. The literature suggests that atypical antipsychotics may contribute to varying degrees to cardiometabolic risk in patients with schizophrenia [14, 15], including aggravation of pre-existing cardiovascular disease and diabetes risk factors [16, 17].

Optimizing antipsychotic treatment for the long-term management of patients with schizophrenia frequently involves the need to switch medication. Over time, patients may be treated with a series of different antipsychotics, with limited evidence-based guidance available to inform clinicians’ choice of the next antipsychotic. In the Clinical Antipsychotic Trials of Intervention Effectiveness (CATIE), which consisted of three 18-month sequential clinical trials in schizophrenia, 74% of participants discontinued their first antipsychotic during the initial phase [18], 69% or 74% discontinued their second antipsychotic in the next phase [19, 20], and 39% discontinued their third antipsychotic in the final phase [21]. Quetiapine and lurasidone are atypical antipsychotics approved by the U.S. Food and Drug Administration (FDA) for the treatment of patients with schizophrenia in 1997 and 2010, respectively [22, 23]. When comparing patients with schizophrenia treated with lurasidone or quetiapine XR, a previously-reported double-blind, clinical trial showed that those treated for 12-months with lurasidone had a statistically significantly reduced risk of hospitalization [24]. The extent to which these findings apply to patients with schizophrenia in real-world treatment settings remains unknown. The primary objective of this study was to confirm and extend these findings by assessment of hospitalization rates among patients with schizophrenia who switched to antipsychotic monotherapy with lurasidone or quetiapine from other atypical antipsychotics in real-world settings. The secondary objectives of this study were to describe treatment duration and total inpatient admission costs for Medicaid and commercial payers.

## Methods

### Study design and database

This retrospective cohort study used U.S. administrative medical and pharmacy claims data from the Truven Health MarketScan® Medicaid Multi-State Database for the period April 1, 2010 through December 31, 2012 and the MarketScan® Commercial Claims and Encounters Database for the period April 1, 2010 through October 31, 2013. Data access was provided based on a licensing agreement between Truven Health and Sunovion Pharmaceuticals Inc. Both databases contain healthcare claims data for patients with Medicaid or employer-sponsored primary insurance. The Medicaid Multi-State Database includes the claims of millions of patients insured by Medicaid from multiple states. Medicaid coverage differs from state to state, and generally includes healthcare coverage for low-income families and individuals as well as individuals with disabilities [25]. The Commercial Claims and Encounters Database includes the claims of patients and their families who are insured through their employers.

**Fig. 1:**
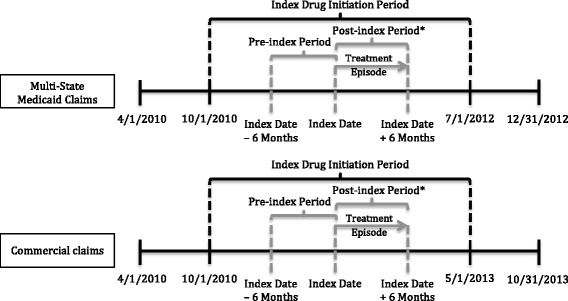
Study design and timeline diagram. The pre-index variables were evaluated from claims during the 6-month pre-index period. The index date was defined as the date of first prescription fill of lurasidone or quetiapine. The outcome variables were evaluated from claims in the 6-month follow-up (post-index) period. Treatment episodes began at, or after, the index date, and could have ended prior to the end of the 6-month follow-up period if the either of the following occurred: treatment discontinuation or a switch to another antipsychotic

All study data were fully compliant with the Health Insurance Portability and Accountability Act (HIPAA) of 1996, and therefore the study did not require approval or waiver from an institutional review board [26]. Please see Fig. 1 for a study design diagram.

### Patient selection criteria

Medicaid and commercial patients were selected separately. All patients included in this study were adults (at least 18 years of age) and diagnosed with schizophrenia (defined by one inpatient or outpatient claim with an International Classification of Diseases, Ninth Revision, Clinical Modification [ICD-9-CM] diagnosis code of 295.xx, where x could be any digit). The 1-year study period was defined as a pre-index period of 6-months prior to the index date, the index date, and a follow-up period of 6-months after the index date. The index date was defined as the date of the first prescription fill of either lurasidone or quetiapine. All patients were required to be continuously enrolled with medical and prescription drug benefits throughout the study period. Patients included in the study were required to switch from another oral atypical antipsychotic (aripiprazole, asenapine, clozapine, iloperidone, olanzapine, paliperidone, risperidone, or ziprasidone) during the pre-index period to antipsychotic monotherapy with either quetiapine or lurasidone.

### Treatment episodes

Treatment episodes were defined by at least two lurasidone or quetiapine prescriptions without overlapping prescriptions for other antipsychotics (atypical, typical, or depot) during the follow-up period. Treatment episodes began on the index date and continued until one of the following: treatment discontinuation (gap of 60 or more days with no index medication), switch to another antipsychotic (initiation of antipsychotic other than the index medication with a gap or overlap of 30 or less days between treatments), or the end of the study’s 6-month follow-up period. Each patient could have multiple treatment episodes. Patients were still included in the analysis if they had switched to a different therapy after one treatment episode ended, and then re-initiated a second treatment episode with either quetiapine or lurasidone monotherapy. The proportion of patients with more than one treatment episode was calculated. Overall treatment duration was defined as the number of days from the start to the end of the treatment episode during the 6-month follow up period.

### Pre-index covariates

Patient pre-index variables were extracted from claims data during the 6-months prior to the index date or on the index date. Psychiatric and cardiometabolic comorbidities were defined based on ICD-9-CM codes (see Additional file 1 for specific codes). Pre-index medications were based on any use of antidepressant, anxiety, hypnotic, mood stabilizer, and stimulant medications as identified using Redbook therapeutic classes. Pre-index atypical antipsychotics included: aripiprazole, asenapine, clozapine, iloperidone, olanzapine, paliperidone, risperidone, and ziprasidone.

### Study outcomes

The main outcome variables were rates of all-cause, mental-health, and schizophrenia-related hospitalizations. Hospitalizations were determined based on the facility claim information. If a patient had facility claims separated by less than one day the claims were considered to be from a single hospitalization. All-cause hospitalization was defined as any hospitalization with any diagnosis during the follow-up period. Mental health-related hospitalization was defined as those with an ICD-9-CM code from 290.xx – 314.xx in the primary or secondary diagnosis position. Schizophrenia-related hospitalization was defined as those with an ICD-9-CM code of 295.xx in any position. Hospitalization rates were examined separately in the Medicaid and commercial payer populations. The proportion of treatment episodes with at least one hospitalization and the length of stay for the first hospitalization were also investigated.

This was a cost of illness study. Due to large differences in payment structures and patient populations, healthcare costs were analyzed separately for the Medicaid and commercial payer populations. Healthcare costs were based on the paid amounts of adjudicated claims, including insurer and health plan payments, as well as patient cost-sharing in the form of copayment, deductible, and coinsurance. Because the most recent year in the Medicaid database was 2012, costs were adjusted for inflation and standardized to 2012 US dollars using the Consumer Price Index from the US Bureau of Labor Statistics [27].

### Statistical methods

Given the high frequency of treatment switching in this population, treatment was studied at the episode level, rather than the patient level. The treatment episode methodology was employed in this analysis to facilitate attribution of hospitalizations to a specific medication [28]. Pre-index characteristics were summarized using means and standard deviations for continuous measures and counts and percentages for categorical measures. Differences between cohorts during the pre-index period were assessed using chi-square tests for categorical variables and t-tests or ANOVA for continuous measures.

For the outcome variables, multivariable generalized linear models were used to statistically adjust for pre-index characteristics while accounting for the within-patient correlation among patients with multiple episodes by using random effect. The models were adjusted for age, gender, payer type, inpatient admissions during the pre-index period, anxiety disorder, depression disorder, alcohol/substance abuse disorder, and anti-anxiety medication use. For probability of resource use, a model with a binomial distribution and logit link was used to compare differences in the probability of hospitalization while controlling for pre-index characteristics. For patients with at least one hospitalization, a model with a gamma distribution [29] with log link was used to compare differences in hospitalization costs while controlling for pre-index characteristics. *P* values <.05 were considered, a priori, to be statistically significant. All analyses were completed with SAS version 9.3 (SAS Institute, Cary, NC).

## Results

### Patient characteristics

**Fig. 2:**
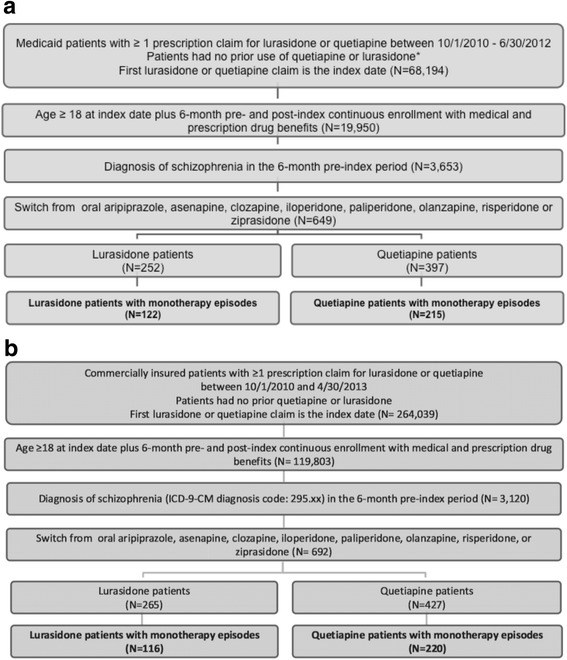
Patients flow through inclusion/exclusion criteria. **a**. Medicaid patient flow diagram. **b**. Commercial patient flow diagram. Abbreviations: ICD-9-CM – International Classification of Diseases, Ninth Revision, Clinical Modification

The database contained 68,194 patients enrolled in Medicaid and 264,039 patients enrolled in commercial insurance during the study period with a first-time prescription for either lurasidone or quetiapine (please see patient flow diagrams, Fig. 2). The number of adults with continuous insurance enrollment with medical and drug benefits throughout the 1-year study period in the database was 139,753 (19,950 Medicaid and 119,803 commercial). Only 3653 Medicaid patients and 3120 commercial patients had a schizophrenia diagnosis (ICD-9-CM diagnosis code 295.xx) during the pre-index period. Of the patients with schizophrenia diagnoses, 649 Medicaid and 692 commercial patients switched from oral aripiprazole, asenapine, clozapine, iloperidone, olanzapine, paliperidone, risperidone, or ziprasidone treatment to lurasidone or quetiapine. Only patients treated with lurasidone or quetiapine as antipsychotic monotherapy were included (337 Medicaid and 336 commercial). Contained in the final sample were 122 Medicaid and 116 commercial patients treated with lurasidone monotherapy, and 215 Medicaid and 220 commercial patients treated with quetiapine monotherapy.

Table 1 provides the patient characteristics for the combined population, as well as for separated Medicaid and commercial payer populations during the pre-index period. In the combined population, lurasidone and quetiapine cohorts were largely similar, with a few notable exceptions. The lurasidone cohort had more females (61% vs. 52%, *p* < 0.05) than the quetiapine cohort. The prevalence of depression (42% vs. 52%, p < 0.05), anxiety (28% vs. 39%, p < 0.05), and alcohol/substance abuse disorder (22% vs. 37%, p < 0.05) was significantly higher for the quetiapine patients than lurasidone patients. While lurasidone patients were more likely to have switched from asenapine or olanzapine, quetiapine patients were more likely to have switched from risperidone (*p* < 0.05). Quetiapine patients also had significantly higher inpatient admission rates during the pre-index period. Overall, multiple monotherapy episodes were uncommon for patients in either the lurasidone (2%) or quetiapine (3%) cohorts. The average daily dose of quetiapine increased significantly during the treatment episodes (mean ± SD first prescription vs. last prescription: 265 ± 256 vs. 306 ± 315 mg *p* < 0.05), while the average daily dose of lurasidone was largely unchanged (mean ± SD first prescription vs. last prescription: 68 ± 105 vs. 74 ± 105 mg, *p* = 0.53).

**Table 1:** Pre-index patient characteristics

Variable	Combined population				Medicaid population				Commercial population			
	Lurasidone		Quetiapine		Lurasidone		Quetiapine		Lurasidone		Quetiapine	
	n = 238		n = 435		*n* = 122		*n* = 215		*n* = 116		*n* = 220	
Demographics												
Age, (Mean, SD)	37.1	13.4	37.6	14.2	39.1	12.8	38.2	12.9	34.9	13.8	36.9	15.3
Female (N, %)	145	61	225*	52	82	67	129	60	63	54	96	44
Comorbidities, (N, %)												
Psychiatric												
Alcohol/Substance Abuse	53	22	159*	37	41	34	84	39	12	10	75*	34
Anxiety	67	28	170*	39	39	32	84	39	28	24	86*	39
Bipolar Disorder	108	45	231	53	57	47	106	49	51	44	125*	57
Depression	99	42	225*	52	51	42	117*	54	48	41	108	49
Personality Disorders	25	11	45	10	18	15	28	13	7	6	17	8
Cardiometabolic												
Diabetes	41	17	61	14	25	21	39	18	16	14	22	10
Hyperlipidemia	48	20	93	21	30	25	52	24	18	16	41	19
Hypertension	69	29	144	33	49	40	88	41	20	17	56	25
Medications, (N, %)												
Psychiatric												
Antidepressants	177	74	313	72	98	80	169	79	79	68	144	66
Anxiety	65	27	172*	40	27	22	78*	36	38	33	94	43
Hypnotics	80	34	142	33	53	43	84	39	27	23	58	26
Mood stabilizers	130	55	269	62	67	55	130	61	63	54	139	6
Stimulants	21	9	22	5	1	1	3	1	20	17	19*	9
Typical antipsychotics	23	10	63	15	12	10	29	14	11	10	34	15
Other												
Antidiabetics	38	16	50	12	22	18	30	14	16	14	20	9
Antihypertensives	62	26	124	29	43	35.	72	33	19	16	52	24
Antilipidemic	55	23	76	18	36	29	40*	19	19	16	36	16
Switch from atypical antipsychotic												
Aripiprazole	58	24	90	21	26	21	41	19	32	28	49	22
Asenapine	24	10	10*	2	12	10	4*	2	12	10	6*	3
Clozapine	4	2	6	1	2	2	3	1	2	2	3	1
Iloperidone	10	4	13	3	5	4	5	2	5	4	8	4
Paliperidone	43	18	74	17	18	15	30	14	25	22	44	20
Olanzapine	28	12	18*	4	17	14	11*	5	11	10	7*	3
Risperidone	56	24	159*	37	27	22	81*	38	29	25	78	35
Ziprasidone	38	16	89	20	23	19	53	25	15	13	36	16
Inpatient admissions, (N, %)												
All-cause	98	41	274*	63	44	36	114*	53	54	47	160*	73
Mental health-related	98	41	272*	63	44	36	113*	53	54	47	159*	72
Schizophrenia-related	80	34	214*	4*	34	28	95*	44	46	40	119*	54
Total cost, (Mean, SD)^a^					13,883	16,721	15,008	14,086	15,379	17,352	23,061*	30,804

^a^Expenditures for the combined Medicaid/Commercial population are not reported due to different payment structures

*p < 0.05; Statistically significantly different from lurasidone cohort

### Hospitalizations during the 6 months follow up period

**Fig. 3:**
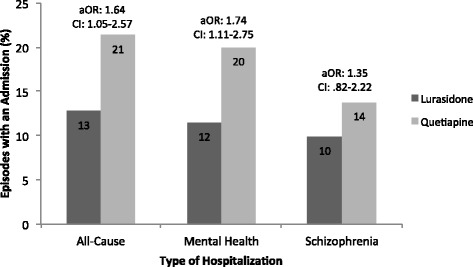
Percent of episodes with at least one all-cause, mental health, or schizophrenia hospitalization in the combined study cohorts. Abbreviations: aOR – Adjusted Odds Ratio, CI – 95% Confidence Interval. Multivariable generalized linear models with binomial distribution and logit link were utilized. The models accounted for within-patient correlation among patients with multiple episodes, and controlled for the variables age, gender, health plan (Medicaid vs. commercial), pre-index hospitalizations, use of anti-anxiety medication, preexisting anxiety, depression, and alcohol/substance abuse

Prior to adjusting for pre-index covariates, lurasidone-treated patients experienced fewer treatment episodes resulting in any hospitalization (13% vs. 21%, p < 0.05), mental health-related hospitalization (12% vs. 20%, p < 0.05), but not schizophrenia-related (10% vs. 14%, *p* = 0.14) hospitalization compared to quetiapine-treated patients. Results from the multivariable generalized linear model that adjusted for pre-index covariates demonstrated that compared to the lurasidone cohort, quetiapine-treated patients had 64% higher odds of all-cause hospitalization (OR = 1.64, 95% CI [1.05, 2.57] *p* = 0.03) and 74% higher odds of mental health-related hospitalization (OR = 1.74, 95% CI [1.11, 2.75], *p* = 0.02), but not schizophrenia-related (OR = 1.35, 95% CI [.82, 2.22], *p* = 0.24) hospitalization in the post-index period (Fig. 3).

Hospitalization rates during the follow up period were higher for patients treated with quetiapine in both the Medicaid and commercial populations, although the differences did not consistently reach statistical significance in the payer subsamples (Table 2). The average length of hospitalization in the Medicaid population appeared to be shorter compared to the commercial population. The average length of stay for the first hospitalization was significantly shorter for Medicaid patients treated with lurasidone than those treated with quetiapine (Table 2).

**Table 2:** Hospitalizations during the 6-month follow up period

Variable	Medicaid population				Commercial population			
	Lurasidone		Quetiapine		Lurasidone		Quetiapine	
	n = 122		n = 215		n = 116		n = 220	
Episodes (N)^a^	125	–	226	–	118	–	223	–
All-cause hospitalizations								
Episodes with ≥1 admission, (N, %)	19	15	52	23	12	10	44*	20
Length of stay (first admission), (Mean, SD)	5.4	4.3	6.7*	4.8	10.9	14.5	9.3	8.8
Cost, among episodes with an admission (Mean, SD)	15,448	13,741	22,012*	23,581	16,265	16,377	27,368*	46,415
Mental health-related hospitalizations								
Episodes with ≥1 admission, (N, %)	18	14	48	21	10	9	42*	19
Length of stay (first admission), (Mean, SD)	5.6	4.4	6.8*	5.0	12.5	15.5	9.8*	8.8
Cost, among episodes with an admission (Mean, SD)	15,388	13,609	22,272*	24,385	16,512	17,903	27,398*	46,476
Schizophrenia-related hospitalizations								
Episodes with ≥1 admission, (N, %)	16	13	36	16	8	7	26	12
Length of stay (first admission), (Mean, SD)	5.6	4.7	7.6*	5.0	14.4	17.0	13.8	21.7
Cost, among episodes with an admission (Mean, SD)	14,193	12,876	21,579*	24,034	17,863	19,745	18,514	17,249

^a^Monotherapy treatment episodes were defined as patients having more than one lurasidone or quetiapine prescription without overlapping other antipsychotics during the post-index period. As some patients had multiple episodes, the N value in this row refers to the number of episodes in the sample that were evaluated

*(*p* < .05); Statistically significantly different from lurasidone cohort

### Treatment duration

Approximately 72% of lurasidone and 68% of quetiapine treatment episodes were censored due to the end of the study period, 12% of lurasidone and 15% of quetiapine discontinued due to a gap of 60 days or more in therapy, and 17% of both lurasidone and quetiapine treatment episodes ended due to switching to another antipsychotic medication. When compared to the duration of quetiapine treatment episodes, lurasidone treatment duration was modestly but significantly longer (mean ± SD: 115.4 ± 49.9 vs. 123.1 ± 47.2 days, *p* < 0.05). Similarly, among those patients who discontinued or switched treatments, duration of lurasidone treatment episodes was longer than quetiapine episodes (mean ± SD: 81.4 ± 39.7 vs. 68.3 ± 33.1 days, p < 0.05).

### Treatment costs

**Fig. 4:**
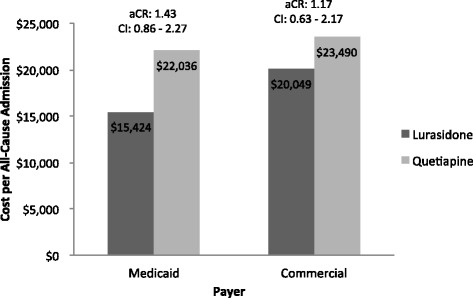
Adjusted all-cause admission costs among hospitalized patients by insurance type. Abbreviations: aCR – Adjusted Cost Ratio, CI – 95% Confidence Interval. Multivariable generalized linear models with a gamma distribution and log link were utilized. The models accounted for the within-patient correlation among patients with multiple episodes, and controlled for age, gender, pre-index hospitalizations, use of anti-anxiety medication, preexisting anxiety, depression, and alcohol/substance abuse, and pre-index total cost. Adjusted costs are based on SAS LS Means

Among treatment episodes resulting in a hospitalization, the unadjusted mean cost of hospitalization for the Medicaid and commercial populations are reported in Table 2. In the multivariable models that adjusted for pre-index covariates (Fig. 4), the adjusted cost of all-cause admissions for those with an admission in the Medicaid population was $15,424 for lurasidone and $22,036 for quetiapine (*p* = 0.17). In the commercial population, the adjusted cost of all-cause admissions for treatment episodes with an admission was $20,049 for lurasidone and $23,490 for quetiapine (*p* = 0.61).

Average total costs, which included hospital admissions, emergency room visits, outpatient services, and prescription costs were computed among all treatment episodes. The average total costs in the post-period were not statistically significantly different between lurasidone and quetiapine (Medicaid: $11,694 vs $12,041, *p* = 0.84; commercial: $7488 vs. $11,158, *p* = 0.14). In the Medicaid cohort, hospital costs made up 20.1% of the total costs for lurasidone treatment and 42.1% for quetiapine treatment, whereas prescription costs made up 32.6% of the total costs for lurasidone treatment and 21.5% for quetiapine treatment. Similarly, hospital costs in the commercial cohort made up 22.1% of the total costs for lurasidone treatment and 48.4% for quetiapine treatment, whereas prescription costs made up 45.9% of the total costs for lurasidone treatment and 18.6% for quetiapine treatment. Additional details of the multivariate results are given in the supplemental online material (Additional file 2).

## Discussion

This real-world analysis of patients with schizophrenia in the U.S., which adjusted for a range of pre-index clinical characteristics and pre-index hospitalizations, found that following a switch from other atypical antipsychotics, treatment with lurasidone was associated with significantly lower rates of all-cause and mental health-related hospital admissions, and similar rates of schizophrenia-related admissions compared with patients switching to quetiapine. In addition, patients switching to lurasidone had significantly longer treatment duration than those switching to quetiapine, potentially due to differences in efficacy or tolerability [30, 31]. The results of this study confirm and extend those of a previously reported prospective trial that demonstrated reduced risk of hospitalization in patients with schizophrenia treated with lurasidone versus quetiapine XR [24]. In the extension study, after 12 months, patients with schizophrenia initially randomized to lurasidone (*n* = 139) had a significantly lower rate of hospitalization than those initially randomized to quetiapine XR (*n* = 79; 9.8% vs 23.1%, *p* = 0.049) [24].

In addition, we found that hospitalization costs associated with lurasidone treatment were lower than with those associated with quetiapine treatment in both commercial and Medicaid populations in this study. Hospitalization costs are the largest contributor to the overall healthcare costs for patients with schizophrenia [32, 33]. They are not only expensive from the perspective of direct costs, but may be an indicator of worse clinical outcomes [34]. These findings suggest that lurasidone may be associated with lower healthcare costs compared with quetiapine and may potentially offer better health outcomes among patients with schizophrenia.

While average daily doses for lurasidone and quetiapine in the current real-world study (74 mg and 306 mg, respectively) were lower than those reported in the extension study [24], these doses were consistent with long-term clinical studies in real-world settings. For example, an average daily quetiapine dose of 377 mg/day was reported in a 3-year prospective study of outpatients with schizophrenia [35], and an average daily lurasidone dose of 87.8 mg/day was reported in a 22-month open-label extension study of patients with schizophrenia [36]. The current study therefore further supports the previous findings that lurasidone may be associated with reduced risk for hospitalization in patients with schizophrenia compared to other atypical antipsychotic agents.

At the time of the study (2010–2013), quetiapine had been available since 1997, while lurasidone had just become available in 2010 [22]. Treatment-resistant patients may be more likely to be prescribed a new medication; therefore, patients who switched to lurasidone may have been more severely ill during the study timeframe. However, only four patients treated with lurasidone and two treated with quetiapine were switched from clozapine, a medication generally reserved for treatment-resistant patients [2]. Nonetheless, there may have been a potential for more severely ill patients to receive lurasidone.

Although atypical antipsychotics significantly reduce the symptoms of schizophrenia, poor treatment duration remains a common clinical problem [18, 37]. Maintaining treatment is essential to achieve optimal clinical benefit, and can reduce the risk of hospitalizations [10, 38]. In this study, treatment duration was modestly, but statistically significantly, longer in the lurasidone cohort than in the quetiapine cohort (123.1 days versus 115.4 days). Treatment options that improve treatment duration are important for the effective management of schizophrenia. The results from this claims analysis, taken together with the results from the long-term prospective double-blind lurasidone versus quetiapine XR clinical trial, suggest that lurasidone may be a more effective treatment than quetiapine for patients with schizophrenia switching from other atypical antipsychotics.

While length of hospital stay appeared to be numerically longer in the commercial population than in the Medicaid population for both lurasidone (14.4 days for commercial vs. 5.6 days for Medicaid) and quetiapine cohorts (13.8 days for commercial vs. 7.6 days for Medicaid) in this analysis, prior national estimates for U.S. schizophrenia-related hospitalizations have showed longer length of stay in Medicaid patients compared to commercial patients (10.9 days for Medicaid vs. 9.1 days for commercial insurance) [39]. The reasons for the differences in the early study and the current study are unclear, but could be due to changes in the composition of beneficiaries or changes in fixed payment rates between payer types that appear to affect length of stay [40].

This study has several limitations inherent to research using administrative claims data. Administrative claims data is collected for reimbursement rather than research purposes. While we have adjusted for many known confounders, other clinical measures that are not available in administrative claims data, may potentially act as additional confounders. Thus the study results, while interesting, may need to be interpreted with caution. For example, the analysis was not able to control for race/ethnicity. In addition, not all patient comorbidities were adjusted for in the analyses. In order to maximize the sample size, patients were included if a single claim with a diagnosis of schizophrenia was present. As with all administrative claims database analyses, medication duration was based on prescription fill dates rather than directly monitored medication administration. Lurasidone and quetiapine are both approved for the treatment of schizophrenia and bipolar depression, but further research would be necessary to determine whether these findings apply to patients with bipolar disorder. Findings from this study pertain to a subset of patients with schizophrenia who switched specific antipsychotics covered under Medicaid or Commercial health plans, thereby limiting the ability to fully extrapolate the findings to all patients. There was no statistical significant difference in schizophrenia-related hospitalization rates, potentially due to the fewer number of events requiring a larger sample size to detect a difference. The direction of the effect for schizophrenia-related hospitalizations matched that for all-cause and mental health-related hospitalizations and the lack of a statistically significance difference does not necessarily imply the lack of a clinical difference. We believe that the commercial data used in this study is representative of the US noninstitutionalized commercially-insured population and that the Medicaid data, which included 12 state Medicaid programs, is representative of all 50 states. The 6-month analytical timeframe was chosen a priori to be sufficiently long to capture hospitalizations related to the study medications. Results of this study are informative about six-months following an antipsychotic switch.

## Conclusions

The results of this real-world analysis suggest that patients with schizophrenia who switched to lurasidone monotherapy had fewer all-cause and mental health-related hospitalizations, but not schizophrenia-related hospitalizations, compared to patients who received quetiapine. Lurasidone treatment was associated with modestly longer duration compared to quetiapine in this study. Given the inverse relationship between treatment duration and hospitalization, it is possible that the higher persistency rates with lurasidone may have contributed to fewer relapses that require hospitalization. These results may inform clinical decision-making as well as value-based decision-making associated with the treatment of schizophrenia.

## Additional files

Additional file 1: ICD-9-CM Diagnosis Codes. (DOCX 52 kb)

Additional file 2: Multivariate Model Results. **Table S1.** Multivariate results for all-cause hospital admission in combined Medicaid and commercial insurance. **Table S2.** Multivariate results for mental-health hospital admission in combined Medicaid and commercial insurance. **Table S3.** Multivariate results for schizophrenia-related hospital admission in combined Medicaid and commercial insurance. **Table S4.** Multivariate results for all-cause hospital costs in commercial insurance among treatment episodes with an admission. **Table S5.** Multivariate results for all-cause hospital costs in Medicaid among treatment episodes with an admission. (DOCX 76 kb)

## Abbreviations

ANOVA: Analysis of variance
FDA: U.S. Food and Drug Administration
HIPAA: Health Insurance Portability and Accessibility Act
ICD-9 CM: International Classification of Disease, Ninth Edition, Clinical Modification
SAS: Statistical Analysis System
SD: Standard deviation
XR: Extended release
