# Supplementary material for: Hospitalization outcomes in patients with schizophrenia after switching to lurasidone or quetiapine: a US claims database analysis
Source: BMC Health Serv Res. 2018 Apr 4;18:243. doi: 10.1186/s12913-018-3020-2 (PMC5885302; doi:10.1186/s12913-018-3020-2)
Supplement: Supplementary file 1 — ICD-9-CM Diagnosis Codes. (DOCX 52 kb) [file 12913_2018_3020_MOESM1_ESM.docx]

Additional file 1

ICD-9-CM Diagnosis Codes

| Condition | Codes |
| --- | --- |
| Alcohol/Substance Abuse | 291.xx, 292.0x, 292.1x, 292.2x, 292.8x, 292.9x, 303.xx, 304.xx, 305.0x, 305.2x -305.9x, 965.xx, 980.0x, E950.0x, V65.42 |
| Anxiety | 293.84, 309.21, 300.0x, 300.2x |
| Depression | 290.21, 300.11, 296.2x, 296.3x, 296.82, 298.0x, 300.4x, 308.0x, 309.0x, 309.1x, 311.xx |
| Personality Disorders | 301.xx |
| Bipolar Disorder | 296-296.06, 296.1-296.16, 296.4, 296.41-296.46, 296.5-296.56, 296.6- 296.66, 296.7, 296.8, 296.81, 296.89, 296.9, 296.99 |
| Hypertension | 401.xx |
| Hyperlipidemia | 272.0x-272.4x |
| Diabetes | 250.xx |
