# Supplementary material for: Hospitalization outcomes in patients with schizophrenia after switching to lurasidone or quetiapine: a US claims database analysis
Source: BMC Health Serv Res. 2018 Apr 4;18:243. doi: 10.1186/s12913-018-3020-2 (PMC5885302; doi:10.1186/s12913-018-3020-2)
Supplement: Supplementary file 2 — Multivariate Model Results. Table S1. Multivariate results for all-cause hospital admission in combined Medicaid and commercial insurance. Table S2. Multivariate results for mental-health hospital admission in combined Medicaid and commercial insurance. Table S3. Multivariate results for schizophrenia-related hospital admission in combined Medicaid and commercial insurance. Table S4. Multivariate results for all-cause hospital costs in commercial insurance among treatment episodes with an admission. Table S5. Multivariate results for all-cause hospital costs in Medicaid among treatment episodes with an admission. (DOCX 76 kb) [file 12913_2018_3020_MOESM2_ESM.docx]

Supplemental Appendix

Multivariate Model Results

Supplemental Table 1. Multivariate results for all-cause hospital admission in combined Medicaid and commercial insurance.

| Variable | Estimate | Odds Ratio | Lower 95% CI | Upper 95% CI | P-value |
| --- | --- | --- | --- | --- | --- |
| Treatment (quetiapine vs. lurasidone) | 0.496 | 1.642 | 1.051 | 2.566 | 0.029 |
| Age (in decades) | 0.199 | 1.220 | 1.050 | 1.419 | 0.009 |
| Gender (female vs. male) | 0.061 | 1.063 | 0.707 | 1.599 | 0.760 |
| Medicaid vs. commercial | 0.389 | 1.475 | 0.968 | 2.249 | 0.071 |
| Prior inpatient admission | 0.949 | 2.582 | 1.579 | 4.222 | <0.001 |
| Anxiety diagnosis | 0.071 | 1.074 | 0.701 | 1.647 | 0.742 |
| Depression diagnosis | 0.218 | 1.243 | 0.822 | 1.878 | 0.303 |
| Alcohol/substance abuse diagnosis | -0.122 | 0.885 | 0.569 | 1.377 | 0.587 |
| Anti-anxiety medication use | -0.198 | 0.820 | 0.532 | 1.264 | 0.369 |

Note. Generalized linear model with binomial distribution, logit link, and a random effect for patient. All variables, except treatment, were coded from the pre-index period.

Supplemental Table 2. Multivariate results for mental-health hospital admission in combined Medicaid and commercial insurance.

| Variable | Estimate | Odds Ratio | Lower 95% CI | Upper 95% CI | P-value |
| --- | --- | --- | --- | --- | --- |
| Treatment (quetiapine vs. lurasidone) | 0.557 | 1.746 | 1.108 | 2.752 | 0.016 |
| Age (in decades) | 0.169 | 1.184 | 1.014 | 1.382 | 0.033 |
| Gender (female vs. male) | 0.232 | 1.261 | 0.830 | 1.916 | 0.277 |
| Medicaid vs. commercial | 0.313 | 1.367 | 0.888 | 2.104 | 0.156 |
| Prior inpatient admission | 0.727 | 2.068 | 1.266 | 3.379 | 0.004 |
| Anxiety diagnosis | 0.205 | 1.228 | 0.799 | 1.888 | 0.348 |
| Depression diagnosis | 0.272 | 1.313 | 0.861 | 2.002 | 0.206 |
| Alcohol/substance abuse diagnosis | -0.035 | 0.966 | 0.615 | 1.516 | 0.880 |
| Anti-anxiety medication use | -0.250 | 0.779 | 0.503 | 1.206 | 0.263 |

Note. Generalized linear model with binomial distribution, logit link, and a random effect for patient. All variables, except treatment, were coded from the pre-index period.

Supplemental Table 3. Multivariate results for schizophrenia-related hospital admission in combined Medicaid and commercial insurance.

| Variable | Estimate | Odds Ratio | Lower 95% CI | Upper 95% CI | P-value |
| --- | --- | --- | --- | --- | --- |
| Treatment (quetiapine vs. lurasidone) | 0.298 | 1.347 | 0.817 | 2.221 | 0.244 |
| Age (in decades) | 0.182 | 1.200 | 1.004 | 1.433 | 0.045 |
| Gender (female vs. male) | 0.200 | 1.221 | 0.765 | 1.948 | 0.402 |
| Medicaid vs. commercial | 0.517 | 1.677 | 1.026 | 2.743 | 0.039 |
| Prior inpatient admission | 0.756 | 2.130 | 1.206 | 3.764 | 0.009 |
| Anxiety diagnosis | 0.290 | 1.337 | 0.821 | 2.176 | 0.243 |
| Depression diagnosis | 0.024 | 1.024 | 0.639 | 1.641 | 0.921 |
| Alcohol/substance abuse diagnosis | -0.111 | 0.895 | 0.530 | 1.512 | 0.679 |
| Anti-anxiety medication use | -0.323 | 0.724 | 0.435 | 1.204 | 0.213 |

Note. Generalized linear model with binomial distribution, logit link, and a random effect for patient. All variables, except treatment, were coded from the pre-index period.

Supplemental Table 4. Multivariate results for all-cause hospital costs in commercial insurance among treatment episodes with an admission.

| Variable | Estimate | Cost Ratio | Lower 95% CI | Upper 95% CI | P-value |
| --- | --- | --- | --- | --- | --- |
| Treatment (quetiapine vs. lurasidone) | 0.158 | 1.172 | 0.633 | 2.167 | 0.614 |
| Age (in decades) | 0.001 | 1.001 | 0.981 | 1.021 | 0.918 |
| Gender (female vs. male) | 0.548 | 1.730 | 1.060 | 2.822 | 0.028 |
| Prior inpatient admission | -0.312 | 0.732 | 0.381 | 1.408 | 0.350 |
| Anxiety diagnosis | 0.127 | 1.135 | 0.667 | 1.932 | 0.641 |
| Depression diagnosis | -0.626 | 0.535 | 0.308 | 0.929 | 0.026 |
| Alcohol/substance abuse diagnosis | 0.533 | 1.704 | 0.935 | 3.105 | 0.082 |
| Anti-anxiety medication use | -0.145 | 0.865 | 0.503 | 1.487 | 0.600 |
| Total cost (in $1000’s) | 0.008 | 1.009 | 1.002 | 1.015 | 0.008 |

Note. Generalized linear model with gamma distribution, a log link, and a random effect for patient. All variables, except treatment, were coded from the pre-index period.

Supplemental Table 5. Multivariate results for all-cause hospital costs in Medicaid among treatment episodes with an admission.

| Variable | Estimate | Cost Ratio | Lower 95% CI | Upper 95% CI | P-value |
| --- | --- | --- | --- | --- | --- |
| Treatment (quetiapine vs. lurasidone) | 0.357 | 1.429 | 0.863 | 2.366 | 0.166 |
| Age (in decades) | 0.042 | 1.043 | 0.988 | 1.021 | 0.622 |
| Gender (female vs. male) | 0.680 | 1.974 | 1.233 | 3.161 | 0.005 |
| Prior inpatient admission | -0.249 | 0.779 | 0.437 | 1.391 | 0.399 |
| Anxiety diagnosis | -0.572 | 0.564 | 0.330 | 0.964 | 0.036 |
| Depression diagnosis | -0.129 | 0.879 | 0.502 | 1.538 | 0.651 |
| Alcohol/substance abuse diagnosis | 0.236 | 1.266 | 0.780 | 2.055 | 0.340 |
| Anti-anxiety medication use | 0.127 | 1.135 | 0.678 | 1.901 | 0.629 |
| Total cost (in $1000’s) | 0.007 | 1.007 | 0.993 | 1.020 | 0.345 |

Note. Generalized linear model with gamma distribution and a log link and a random effect for patient. All variables, except treatment, were coded from the pre-index period.
